# Eosinophilic granulomatous gastrointestinal and hepatic abscesses attributable to basidiobolomycosis and fasciolias: a simultaneous emergence in Iraqi Kurdistan

**DOI:** 10.1186/1471-2334-13-91

**Published:** 2013-02-20

**Authors:** Hemmin A Hassan, Runnak A Majid, Nawshirwan G Rashid, Bryar E Nuradeen, Qalandar H Abdulkarim, Tahir A Hawramy, Rekawt M Rashid, Alton B Farris, Jeannette Guarner, Michael D Hughson

**Affiliations:** 1 Departments of Pathology, Medicine, and Surgery, Shorsh General Hospital, Sulaimaniyah, Iraq; 2 Departments of Microbiology and Surgery, Sulaimaniyah University College of Medicine, Sulaimaniyah, Iraq; 3 Ministry of Health, Kurdish Regional Government, Erbil, Iraq; 4 Department of Pathology, Emory University, Atlanta, GA, USA

**Keywords:** Granulomas, Eosinophilia, Fascioliasis, Basidiobolomycosis, Gastrointestinal infections

## Abstract

**Background:**

Deep eosinophilic granulomatous abscesses, as distinguished from eosinophilic subcutaneous abscesses, are rare. Most reports are from the Far-East and India where the most commonly attributed cause is *Toxocara*. Sulaimaniyah in Northeastern Iraq has experienced an outbreak of eosinophilic granulomatous liver and gastrointestinal (GI) abscesses beginning in 2009. The purpose of this study was to determine the etiology and guide treatment.

**Methods:**

The study was an ongoing investigation of patients having a histopathologic diagnosis of eosinophilic granulomatous abdominal abscesses in Sulaimaniyah hospitals from May 2009 to August 2012. Tissues were examined for organisms, and Enzyme Linked Immunoabsorbent Assays (ELISA) were performed for serum antibodies to *Fasciola hepatica*, *Toxocara*, and *Echinococcus granulosus.*

**Results:**

Fourteen patients had granulomatous inflammation surrounding a central necrotizing eosinophilic exudate identified in surgical pathology specimens from abdominal surgeries. Two children and four adults had abscesses that formed GI masses. These patients included a 39 year old male with oropharyngeal and transverse colon disease, and a 48 year old male with liver and GI abscesses. All sites demonstrated a Zygomycete fungus surrounded by eosinophilic Splendori-Hoeppli material consistent with basidiobolomycosis. Five of the six patients with fungal disease were treated by surgery and 4 to 7 months of itraconozol. One child died of intestinal perforation while receiving IV amphotericin B; two adults required additional surgery for recurrent GI obstruction. Eight patients had isolated liver abscesses with no organisms identified by histopathology: ELISA results for *F. hepatica* were positive for five, borderline for one, and negative for two patients. These eight patients were treated for fascioliasis by surgical resection of localized abscesses and albendazol. One patient serologically positive for *F. Hepatica* was found to have a common duct fluke two years after initial diagnosis. Serological testing for *Toxocara* and *Echinococcus granulosus* was negative in all 14 patients.

**Conclusions:**

Basidiobolomycosis and *F. hepatica* are implicated as the cause of abdominal eosinophilic granulomatous abscesses in 12 of 14 patients identified over a period of 40 months in northern Iraq. Treatment was complicated by chronic biliary tract disease in fascioliasis and perforation and recurrent intestinal obstruction with basidiobolomycosis.

## Background

Eosinophilic and granulomatous liver and gastrointestinal (GI) abscesses, as distinguished from Langerhans cell disease, are barely mentioned in the pathology textbooks [1-6]. A few articles are available from the Far East, South America, and India where *Toxocara* as part of visceral larvae migrans is implicated, although often not proven, as being the etiologic agent [7-12]. In 2001, eosinophilic hepatic granulomas were reported in 43 cases from the files of the Armed Forces Institute of Pathology (AFIP) that were collected over 31 years [13]. *Toxocara* was identified in 30% of the specimens and was suggested to be the causative organism for the series. Based upon the experience with autopsies in Uruguay, Vercelli-Retta et al. [14,15] in a following letter to the editor cautioned that *Fasciola hepatica* may be involved in similar cases, if not actually some of those in the AFIP collection. The fungal disease basidiobolomycosis is an additional cause of eosinophilic granulomatous gastrointestinal (GI) abscesses [16-19]. GI basidiobolomycosis affects the colon or rectum in more than 80% of cases, but small intestinal or liver involvement are each found in 30% of patients [19].

The liver fluke, *F. hepatica*, is one of the most widespread of all parasites that can be transmitted to humans [20]. It is found on all continents except Antartica, and is a major veterinary problem with an important economic impact on the animal reservoirs of sheep, goats, and cattle [20-22]. Since the 1980s, human infections have been increasingly identified with highly endemic areas being recognized in the American Andes, the Nile Delta, and Iran [20-26]. Outside of these endemic regions, human fascioliasis is uncommon, but outbreaks have been reported from Portugal and France [25,27]. *F. hepatica* infestations have an acute and a chronic phase [28]. In the acute stage, immature flukes penetrate the small intestinal wall and migrate into the liver. Most of these immature organisms are destroyed, but after 3–4 months, those that survive mature into adult flukes and inhabit the biliary tract where they can live for more than a decade [28].

GI basidiobolomycosis is a rare but increasingly recognized infection caused by the fungus *Basidiobolus ranarum*[16]. Vikram et al. [19] reviewed the worldwide cases reported through March 2010 and identified 44 patients diagnosed with GI basidiobolomycosis. The largest numbers of cases came from the desert climates of Saudi Arabia (11 patients) and Arizona in the Southwestern United States (17 patients), but four patients each were reported from Brazil and Iran and single patients from Nigeria, India, Bangladesh, Italy, the Netherlands, and an unknown country [16-19]. Recently, 14 additional patients were reported from Shiraz, Iran, a city with a climate and geography similar to Sulaimaniyah [29]. The fungus occurs worldwide, and in the tropics, subcutaneous eosinophilic abscesses due to the traumatic inoculation of basidiobolomycosis are well recognized [16]. The source of the GI infections is not known, but pica and eating unwashed vegetables have been suggested as predisposing factors [30].

Sulaimaniyah is the provincial capital of the eastern region of Northern Iraq. Since the 2003 Gulf War, the city has grown rapidly and has incorporated pastoral and agricultural lands where cattle, sheep, and goats are raised together with fruit and vegetable crops in close proximity to newly developed neighborhoods. The mountain highlands of Northern Iraq have a semi-arid summer climate much like the Southwestern United States but with cool, wet winters similar to endemic areas of *F. hepatica* in the Andes [28].

Because deep eosinophilic granulomatous abscesses have different causes according to the part of the world from which they are reported, a cluster of such abdominal abscesses occurring over a period of 40 months in Sulaimaniyah, Iraq were investigated by serological and tissue studies to identify possible infectious agents. The findings are presented to provide physicians, surgeons, and pathologists an awareness of deep eosinophilic abscesses and to report our etiologic findings from this region of the Middle-East.

## Methods

The Sulaimaniyah Directorate of Health serves as the ethics committee for Shoresh General Hospital and gave permission for the research. The research was conducted according to the Helsinki Accords. Written informed consent was obtained from patients and, in the case of children, from parents for participation in the study. Consent was granted for using personal information and specimen and radiographic images as case reports.

The study is a clinicopathological evaluation of patients in which eosinophilic granulomatous inflammation not attributable to Langerhans cell disease was recognized in surgical pathology specimens. From May 2009 to August 2012, fourteen patients were identified by pathologists (HAH, RAM, and MDH) as having liver or gastrointestinal abscesses composed of eosinophilic granulomatous inflammation. The surgical pathology specimens were received in 10% formaldehyde in the pathology departments of the two major Sulaimaniyah hospitals, Shorsh General Hospital and the Teaching Hospital of the University of Sulaimaniyah. None of the tissue was received fresh and neither the pathologists nor the surgeons submitted any specimens for bacterial or fungal cultures. The gross specimens of liver were cross-sectioned at 3–4 mm intervals and examined grossly for larvae, round worms, or flukes. For microscopy, 4–16 blocks of tissue were obtained from the formalin-fixed gross specimens and serial sections were trimmed from the paraffin blocks. The slides were stained with hematoxylin and eosin and periodic acid-Schiff-hematoxylin (PAS-H) stains. Microscopic slides were reviewed by all pathologists (HAH, RAM, ABF, JG, and MDH).

With the identification of the early cases, the pathology was recognized as being unusual, and beginning in May of 2010 when fungal elements were first seen, microscopic slides and paraffin blocks were sent for consultation to experts in infectious disease pathology at Emory University, Atlanta, GA (ABF, JG). One of the Atlanta pathologists (JG) had been involved in evaluating the Arizona basidiobolomycosis cases [16].

All patients had abdominal ultrasound examinations, and 9 of the 14 patients had abdominal computerized tomography (CT). Three patients with abdominal abscesses (patients 1, 3, and 5) had endoscopy and endoscopic colon biopsies before laparotomy and surgery. A standardized questionnaire was developed, and patients were interviewed about symptoms and their time of onset, place of residence, type of employment, diet, contact with animals, and sources of food. Interviews were conducted after the histopathologic diagnosis were made, and after May 2010, before the initiation of anti-fungal or anti-parasitic therpy. Prior to May of 2010, we did not have kits for serological testing, and based upon our review of the literature, three patients (7, 13, and 14) were suggested to have *Toxocara* infestations and were treated with albendazol. They were later recalled for serologic tests.

At the time of the interviews, preoperative medical records were reviewed, blood samples were collected for completely blood counts and serological studies, and stool samples were examined microscopically for ova and parasites with emphasisis on the ova of *Fasciola*. Enzyme Linked Immunoabsorbent Assays (ELISA) were performed using commercial kits for *F. hepatica* (Pishtaz Teb Zaman™ Diagnostics, Tehran, Iran), *Echinococcus* (Pishtaz Teb Zaman™ Diagnostics, Tehran, Iran), and *Toxocara* (Diagnostic Automation™, Inc, Calabasa, CA). All kits detected IgG antibodies to the indicated parasites using solid phase excretory-secretory (ES) antigens. All tests were performed in duplicate with positive and negative controls.

Irbil and Dohuk are the other large cities in the Kurdish region that have medical reference centers. Irbil lies in the center of the region and Duhuk in the West near the Syrian border. HAH, RAM, and MDH have had several discussions with the pathologists at the Hawler University Teaching Hospitals in Irbil and the Teaching Hospital of Dohuk University about whether they have encountered similar cases in their Directorates with the latest queries being by telephone in December of 2012

To find a possible source of the organisms, cattle were investigated at the regional slaughterhouse in October 2010 by the senior consultant pathologist of Shorsh Hospital (MDH). The livers, biliary tracts, and upper gastrointestinal tracts of cattle processed during one day of work were examined by serially cross sectioning the livers and opening the duodenums and common bile ducts.

## Results

A total of 14 patients (nine males and five females) were identified as having eosinophilic granulomatous abdominal abscesses. Two of the males were 18 month old children. The median age of the adults was 36 with a range of 17 to 59 years old. Summaries of patient clinical, radiological, and laboratory characteristics are provided in Table 1 and pathology, treatment, and follow-up information in Table 2. None of the patients were diabetic or were being treated with corticosteroids or other immunosuppressive drugs. HIV infections are very rare in the Kurdish region, and none of the patients had any HIV risk factors or had traveled outside of Iraq. Stools were negative for ova and parasites.

**Table 1:** Clinical, radiologic, and laboratory characteristics of 14 patients diagnosed with granulomatous and eosinophilic gastrointestinal and hepatic abscesses in Sulaimaniyah, Iraq, 2009–2012

**Patient**	**Age (yrs)**	**Sex**	**Date (mo/yr) and method of diagnosis**	**Social and medical history**	**Febrile**	**Weight loss**	**Eosino-philia**	**Principal symptoms/duration**	**Radiologic findings**	**Fasciola ELISA**
1.	48	M	5/2010; histology	Cashier/healthy	Yes	Yes	12%	Abdominal pain/2 mos, intestinal mass, obstruction	CT: Cecal, ascending colon mass, stricture. Rt. lobe liver mass	Neg
2.	1.5	M	8/2010; histology, biopsy of pelvic mass	Previously well infant	Yes	Yes	29%	Abdominal pain, pelvic mass/1 wk.	CT: Necrotic mass, lower abdomen and pelvis	Neg
3.	59	M	10/2010; histology	Merchant, dry goods/healthy	Yes	Yes (6 kg)	No	Abdominal pain and mass. Itching/3 mos.	CT: Cecal mass	Neg
4.	53	M	2/2012; histology	Soldier/healthy	Yes	Yes	Not tested	Abdominal pain/1 mo., intestinal mass, obstruction	CT: Cecal, transverse colon masses, strictures	Neg
5.	39	M	5/2012; histology	Military office worker/chronic gastritis	Yes	Yes	9%	Persistent cough, sore throat/1 mo.	CT: Diffuse soft tissue mass of oropharynx. Transverse colon mass, stricture	Neg
6.	1.5	M	8/2012; histology	Previously well infant	Yes	Yes	21%	Abdominal pain/2-3 d, intestinal mass, obstruction	CT: Cecal mass, thick ascending colon with stricture	Neg
7.	30	M	6/2009; histology, serology	Janitor/healthy	Yes	Yes	26%	R subcostal pain/1 mo.	CT: Cystic mass Rt. lobe liver. Mild splenomegaly	Pos
8.	30	F	5/2010; histology, serology	Housewife/healthy	No	No	> 50%	R subcostal pain/1 wk.	US: Small hypodense liver lesions. Mild splenomegaly	Pos
9.	56	F	6/2010; histology, serology	Housewife/healthy	No	Yes (7 kg)	35%	R subcostal pain/1-2 wks.	US: Small hypodense liver lesions	Pos
10.	56	F	8/2010; histology, serology	Housewife/healthy	Yes	Yes	26%	R subcostal pain/1-2 wks.	US: Cystic mass Rt. lobe liver	Pos
11.	33	M	11/2011; histology, serology	Shopkeeper/healthy	Yes	Yes (10 kg)	72%	R subcostal pain/2-3 wks.	CT: Cystic mass Rt. lobe liver. Mild splenomegaly	Pos
12.	22	F	5/2010; histology, serology	Student/healthy	No	Yes (4 kg)	74%	R subcostal pain/1 wk.	US: Small hypodense liver lesions. Mild splenomegaly	Borderline
13.	17	F	5/2009; histology	Student/healthy	Yes	Yes	23%	Abdominal pain/2-3 wks.	US: Cystic mass Rt. lobe liver. Mild splenomegaly	Neg
14.	32	M	4/2009; histology	Soldier/healthy	No	No	29%	R subcostal pain/1-2 mos.	CT: Cystic mass Rt. lobe liver	Neg

Abbreviations: *M* male, *F* female. Mo, month; wk, week; d, day. CT, computerized tomography; US, ultrasonography. Neg, negative; Pos, positive.

**Table 2:** Pathological findings, treatment, and follow-up for 14 patients diagnosed with granulomatous and eosinophilic gastrointestinal and hepatic abscesses in Sulaimaniyah, Iraq, 2009–2012

**Patient**	**Surgical procedure**	**Pathologic findings and location of disease**	**Size of abscesses**	**Organism in tissue**	**Medical treatment**	**Complications**	**Follow-up duration**
1.	Right hemicolectomy Segmental hepatectomy	Cecal, ascending colon, and localized liver masses	8-12 cm	Pauci-septate hyphae	Oral itraconozol, 6 mos.	Recurrent obstruction after 5 mos.	Asymptomatic,24 mos.
2.	Biopsy of mass	Pelvic mass, intestinal site uncertain	9 cm	Pauci-septate hyphae	IV amphotercin B, 2 d.	Died	
3.	Right hemicolectomy	Cecal mass	6 cm	Pauci-septate hyphae	Oral itraconozol, 6 mos	None	Asymptomatic, 26 mos.
4.	Right hemi and transverse colectomy	Cecal and transverse colon masses	17 cm	Pauci-septate hyphae	Oral itraconozol, 6 mos.	Recurrent obstruction after 3 mos.	Asymptomatic, 10 mos.
5.	Transverse colectomy	Oropharyngeal and transverse colon masses	20x9 cm	Pauci-septate hyphae	Itraconozol: IV14 d, Oral 7 mos	None	Asymptomatic, 7 mos.
6.	Right hemicolectomy	Cecal mass	4 cm	Pauci-septate hyphae	Oral itraconozol, 4 mos.	None	Asymptomatic, 4 mos.
7.	Segmental hepatectomy	Liver mass, localized	4x6 cm	No	Albendazole 400mg/d, 3 d	None	Asymptomatic, 41 mos.
8.	Cholecystectomy, wedge liver biopsy	Liver, diffuse abscesses	0.5-2 cm	No	Albendazole 400 mg/d, 3 d	None	Asymptomatic, 30 mos.
9.	Cholecytectomy, wedge liver biopsy	Liver, diffuse abscesses	0.5-2 cm	No	Albendazole 400 mg/d, 3 d	Common duct fluke after 2 yrs	Asymptomatic, 30 mos.
10.	Segmental hepatectomy	Liver mass, localized	3 cm	No	Albendazole 400 mg/d, 3 d	None	Asymptomatic, 28 mos.
11.	Segmental hepatectomy	Liver mass, localized	5 cm	No	Albendazole 400 mg/d, 3 d	None	Asymptomatic, 13 mos.
12.	Cholecytectomy, wedge liver biopsy	Liver, diffuse abscesses	0.5-2 cm	No	Albendazole 400 mg/d, 3 d	None	Asymptomatic, 30 mos.
13.	Segmental hepatectomy	Liver mass, localized	4x5 cm	No	Albendazole 400 mg/d, 3 d	None	Asymptomatic, 42 mos.
14.	Segmental hepatectomy	Liver mass, localized	2 cm	No	Albendazole 400 mg/d, 3 d	None	Asymptomatic, 43 mos.

Abbreviations: Mo, month d, day.

Patient 1 lived in a rural mountain village to the North of Sulaimaniyah; all other patients lived in urban Sulaimaniyah and all had visited rural areas multiple times within the year prior to diagnosis. Only patient 5 had any significant previous disease and had received ongoing treatment for chronic gastritis with esomeprazole beginning at age 25. None of the patients had occupations related to farming, produce distribution, or animal care. All patients ate market vegetables that were washed with water or light soap or salt solutions. Vinegar or potassium permanganate was not used for washing vegetables by any of the patient’s families.

**Figure 1:**
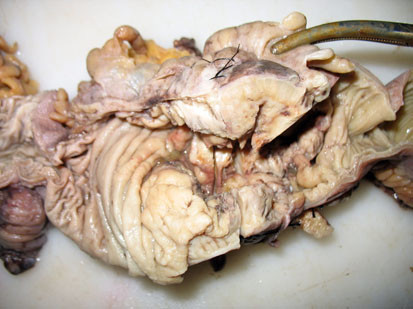
**Patient 1.** A right hemicolectomy specimen showing a cecal mass with irregular mucosal ulceration.

**Figure 2:**
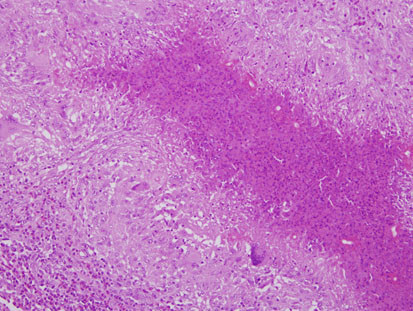
**Patient 1.** The wall of the cecum demonstrates a stellate abscess with a necrotic center containing eosinophils surrounded by granulomatous inflammation with Langerhans giant cells. Fungal elements can be seen surrounded by an eosinophilic Splendore-Hoeppli reaction. Hematoxylin and eosin stain ×100.

**Figure 3:**
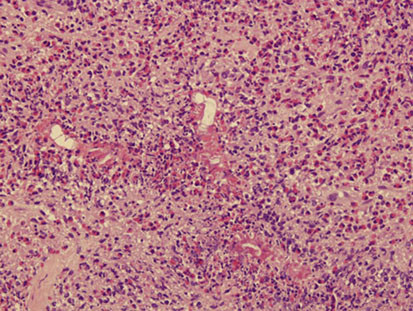
**Patient 5.** The microscopy of a tonsilar biopsy reveals an intense eosinophilic inflammatory reaction containing fungal hyphae surrounded by the Splendore-Hoeppli phenomenon. Hematoxylin and eosin stain ×200.

**Figure 4:**
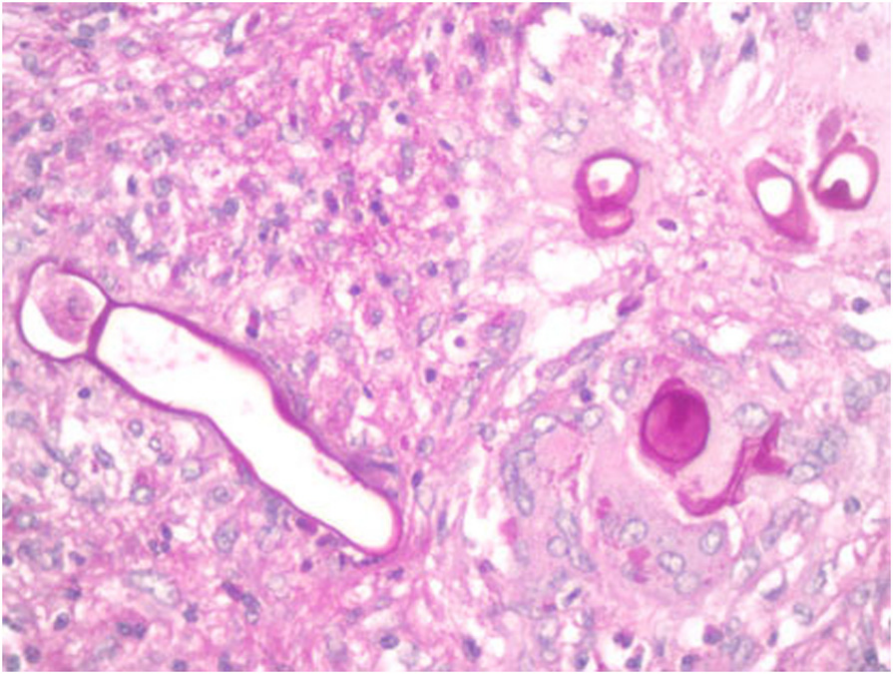
**Patient 1.** The fungi consist of broad pauci septate hyphae consistent with basidiobolomycosis. Periodic acid-Schiff hematoxylin stain ×400.

**Figure 5:**
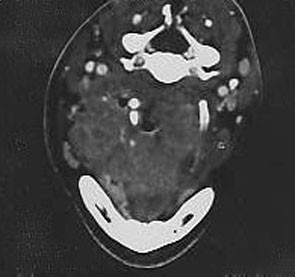
**Patient 5.** Magnetic resonance imaging showing a mass at the base of the tongue that markedly narrows and shifts the oropharynx laterally.

Six patients had GI ulcers that in four patients produced tumor-like cecal and ascending colon masses composed of eosinophilic abscesses (Figure 1). The abscesses had irregularly shaped necrotic centers containing eosinophils with variable numbers of neutrophils (Figure 2). The margins of the abscesses were lined by a granulomatous reaction consisting of epithelioid histiocytes and Langerhan’s giant cells. Eosinophil crystals were occasionally but not commonly seen. The abscesses and surrounding inflammation contained broad pauci-septate hyphae surrounded by eosinophilic material producing the Splendore-Hoeppli phenomenon (Figure 3). The hyphae were PAS positive and morphologically resembled the Zygomycete fungus *Basidiobolus ranarum* and were consistent with a diagnosis of probable basidiobolomycosis (Figure 4). These patients included a 39 year old man with ulcerating eosinophilic granulomatous inflammation that produced extensive soft tissue infiltration that narrowed the nasopharynx and involved the oral cavity including the floor of the mouth, base of the tongue, supraglottis, and glottis (Figure 5). The severe stenosis of the laryngopharynx required a tracheostomy. Three months after the diagnosis of the oropharyngeal disease, the patient developed intestinal obstruction and was found to have an eosinophilic granulomatous mass in the transverse colon that contained hyphae with a Splendore-Hoeppli reaction. One of the patients with GI disease also had a localized liver abscess containing fungus.

**Figure 6:**
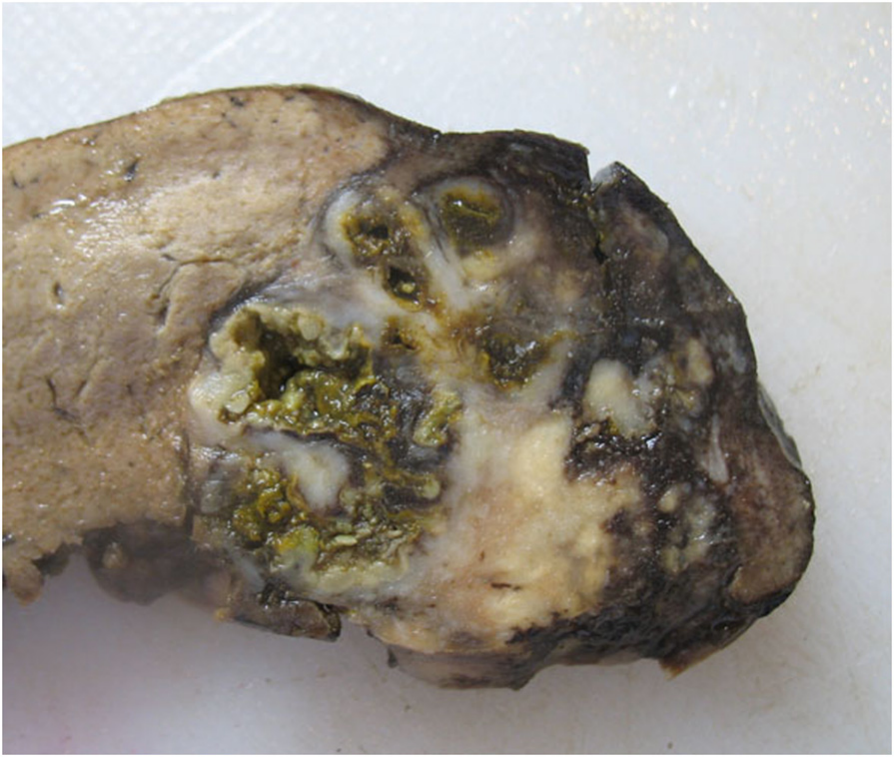
**Patient 7.** Localized abscess of the right lobe of the liver in a patient serologically positive for *F. hepatica*.

**Figure 7:**
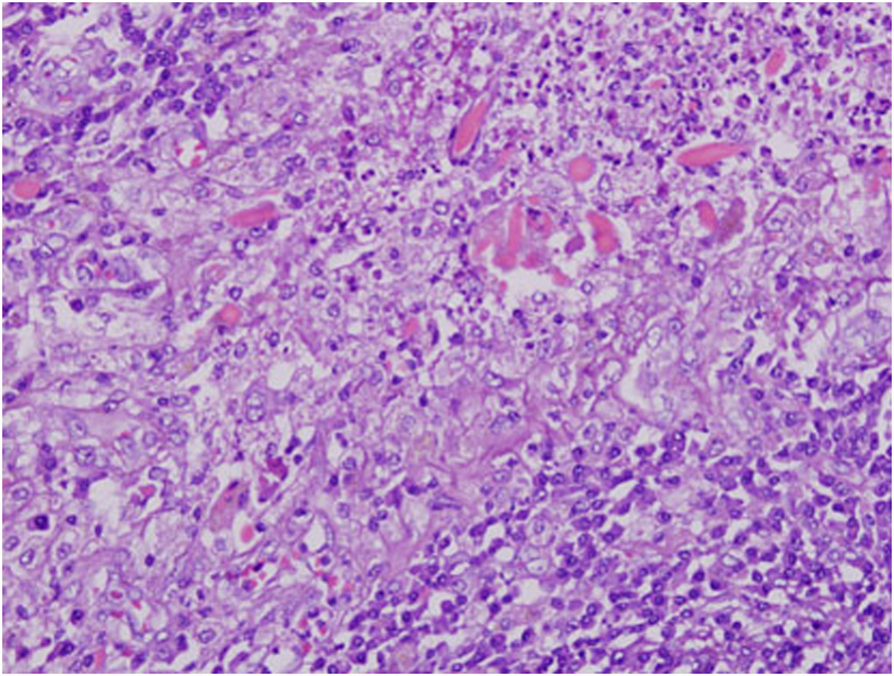
**Patient 7.** The abscess consists of a peripheral rim of epithelioid histiocytes and central necrosis with neutrophils and eosinophils. Numerous eosinophil crystals are present. Hematoxylin and eosin ×400.

Eight patients had isolated eosinophilic granulomatous liver abscesses. In 5 patients the disease was localized with a single multilocular lesion being identified radiologically and at laparotomy in the right hepatic lobe (Figure 6). Localized abscesses were resected by segmental hepatic lobectomy. In 3 patients, the disease was diffuse with many small lesions (0.5-2 cm) being scattered throughout the liver. The patients with diffuse small abscesses were operated on for symptoms of gall bladder disease, and underwent cholecystectomy and wedge biopsies of the liver abscesses. The gall bladders showed mild chronic cholecystitis, and by ultrasonography, the common ducts were not dilated and contained no stones or shadows. The isolated liver abscesses, both localized and diffuse, consisted of epithelioid and mutinucleated histiocytes surrounding irregular central zones of necrotic neutrophils and eosinophils with prominent elongate or rhomboid eosinophil crystals (Figure 7). Neither larvae suggesting *Toxocara* nor *Fasciola* flukes or ova were found in any of the liver specimens.

Five of the 8 patients with isolated liver abscesses tested serologically positive for *F. hepatica,* one had a borderline result, and two were negative. The two negative patients had their disease identified before May of 2010 and had been treated with albendazol before having blood drawn for serological testing 14 and 15 months after diagnosis. One serologically positive patient had blood drawn 13 months after being treated. All six patients with probable basidiobolomycosis had negative fasciola ELISA results. All 14 patients were serologically negative for *Toxocara* and *Echinococcus*.

The adults and one child with GI basidiobolomycosis were treated by right hemicolectomy and/or transverse colectomy for the GI masses, and for the patient with liver involvement by segmental hepatectomy. Surgery was followed by oral itraconozol at 400 mg/day for 4 to 7 months. Two adults required additional surgery for recurrent intestinal obstruction, one at 3 months and the other at 5 months following the original colonic resection. One of the children (patient 2) developed intestinal perforation and died while he was receiving intravenous amphotericin B. Intravenous itraconozol was not available, and the child was too ill to undergo extensive surgery at the time basidiobolomycosis was diagnosed on the basis of an incisional biopsy obtained at laparotomy from the outer aspect of a large pelvic mass. Intravenous itraconozole was obtained for the patient with oropharyngeal disease. After two months, the tissues of the oropharynx decreased in size, and airway and esophageal access were restored. The infected segment of transverse colon, that was found three months after beginning treatment of the oropharyngeal disease, was resected, and the patient continued 7 months of oral itraconozle with no further complications.

After surgery, patients with isolated liver abscesses including those with positive, borderline, and negative fasciola ELISA results were treated with a 3 day course of 400 mg daily of albendazole. Constitutional symptoms resolved within the 3 day period of treatment and eosinophilia within one to 3 months afterward. Two years after her initial treatment, patient 10 developed right upper quadrant pain and tenderness and was found to have a dilated common duct with a moving shadow by ultrasonography. A fluke was extracted from her common bile duct by ERCP. She was then given a single dose of 500 mg of triclabendazole and has since been asymptomatic and has had no eosinophilia.

Most of the patients came to medical attention during the warmer and dryer months of the year. The patients with probable basidiobolomycosis were diagnosed in February, May (2 patients), August (2 patients), and October. Patients serologically positive or borderline for *F. hepatica* were diagnosed in May (one positive, one borderline), June (2 patients), August, and November. The two patients with isolated liver abscesses and negative fasciola serology were diagnosed in April and May.

Since beginning the study, we have had one case of cutaneous chromoblastomycosis and two cases of aspergillus allergic fungal sinusitis, but have not seen any eosinophilic subcutaneous abscesses nor any subcutaneous or sinus fungal infections consistent with entomophthoromycosis. We have had one case of invasive sino-nasal mucormycosis in a diabetic. Cancer chemotherapy patients are usually investigated by bronchial washings or fine needle aspiration cytology rather than biopsies for pulmonary infections. With this limitation, we have had only two bronchial washes containing *Candida* as potential fungal pulmonary infections. Endoscopic colon biopsies before laparotomy on patients 1, 3, and 5 showed eosinophilic granulomatous inflammation; fungi were seen only in the biopsy of patient 5. One eosinophilic liver abscess was reported at the Teaching Hospital of Duhuk University in 2010; none have been seen by the pathologists at Hawler University Teaching Hospitals in Erbil.

**Figure 8:**
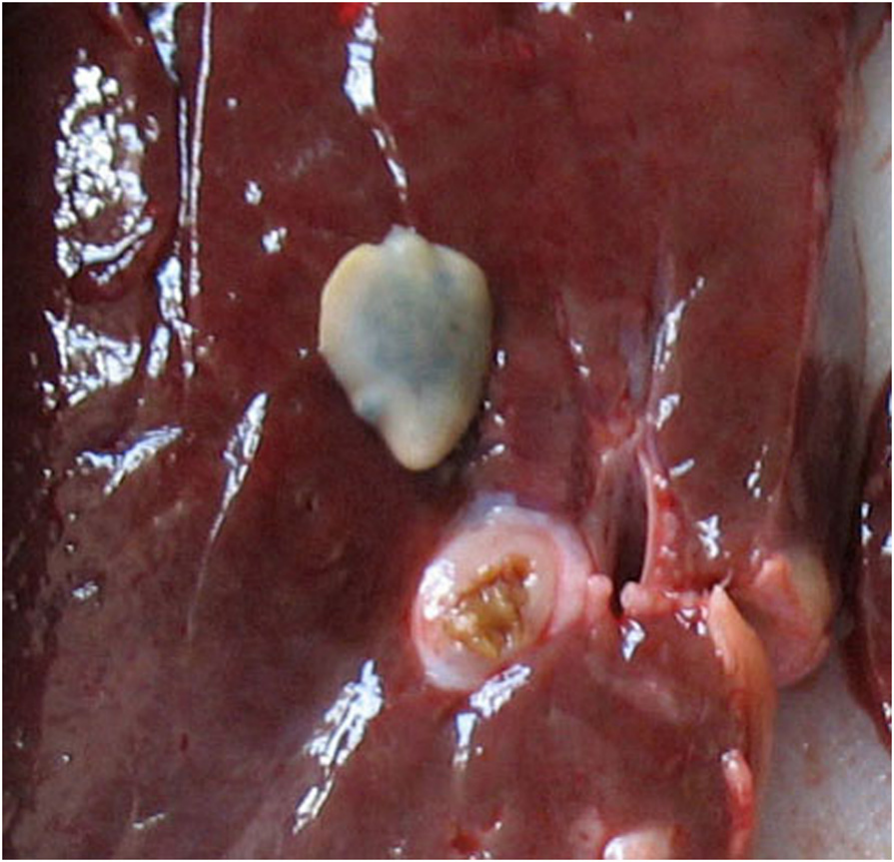
**Liver of a bull slaughtered at the regional abattoir showing an adult fluke of *F. hepatica *that was found in the thickened bile duct below the parasite.**

Thirty seven cattle were examined at the Sulaimaniyah slaughterhouse and *F. hepatica* was identified in ten animals (27%). Immature flukes were found in the duodenums, and adult flukes were identified in thickened intrahepatic bile ducts (Figure 8). None of the cattle showed any evidence of fungal disease.

## Discussion

In this article, we report from Northern Iraq a cluster of GI and hepatic eosinophilic granulomatous abscesses that were not recognized in the region prior to 2009. This pathology is relatively rare, and the medical literature is vague about possible causative agents. We show that, in this region of Iraq, some eosinophilic granulomatous abscesses can be attributed to *F. hepatica* and others to a fungus consistent with *Basidiobolus ranarum*. Although, two of the 14 cases could not be ascribed to a specific etiologic agent, none of the patients had serological or pathological evidence for *Toxocara* or *Echinococcus granulosis*, the latter being the most common clinical parasitic disease of the region [31,32].

The currently recommended criteria for the diagnosis of fascioliasis include peripheral eosinophilia and positive serological testing for antibodies to the organism [33,34]. Additional symptoms of the acute hepatic stage consist of fever, malaise, right subchondral pain, and weight loss. CT may show irregular hypodense lesions within the liver [35]. Stool examinations for eggs can be diagnostic but are usually negative because of a generally low concentration of adult flukes and irregular shedding of eggs [34]. The immature flukes that penetrate the liver are rarely seen in histologic sections, and a tissue diagnosis of fascioliasis is usually not possible [15,28].

On the basis of the serological findings, five of the 14 patients with eosinophilc abscesses were diagnosed as having *F. hepatica* infestations. On the basis of a borderline serological result, one additional patient was considered to be suspicious for fascioliasis. All 6 of these patients presented with right subcostal abdominal pain and eosinophilia. By CT or ultrasonography, a 3 to 6 cm in diameter cystic mass was found in the right lobe of the liver in 3 patients, and small hypodense lesions were scattered thoughout the liver in 3 other patients. The surgical pathology specimens of both the larger and the smaller lesions demonstrated eosinophilic granulomatous abscesses with no evidence of flukes or ova.

In six patients, the eosinophilic abscesses contained pauci-septate hyphae surrounded by a Splendore-Hoeppli reaction. While a definitive diagnosis requires culture, a probable diagnosis of basidiobolomycosis can be made histologically [30]. The species *Basidiobolus ranarum* is a *Zygomycete* fungus belonging to the order *Entomophthorales*[36]. The differential diagnosis consists of the entomophthoromycosis *Conidiobolus coronatus* and *Conidiobolus incongruous* that like basidiobolomycosis incites an eosinophilic granulomatous tissue reaction and the Splendore-Hoeplli phenomonon. *Conidiobolus* is primarily a tropical disease that produces sinonasal infections and subcutaneous abscesses. Oropharyngeal and laryngeal disease has been reported, but *Conidiobolus* is not a known cause of intestinal infections [36]. Vikram et al. [19] have recommended that the definition of probable GI basidiobolomycosis include the identification of disease at a site below the esophagus in the stomach, large or small intestine, anus, liver, gall bladder, or pancreas.

In this current study, patients with probable basidiobolomycosis had fever, malaise, weight loss, and mild to moderate eosinophilia, but the predominant finding was an abdominal mass with intestinal obstruction. One patient had an ulcerating oropharyngeal and glottic mass and obstructing disease of the transverse colon. This oropharyngeal disease was included as an example of GI basidiobolomycosis on the basis of the patient's colon involvement and on the basis of the definition of the GI tract by the National Library of Medicine-Medical Subject Headings including all derivatives of the embryonic entoderm from the mouth to the anus [37]. As an example of probable oropharyngeal basidiobolomycosis, the case extends the reported level of GI involvement to above the stomach and overlaps with locations infected by *Conidiobolus*[19,36].

It is suggested that GI basidiobolomycosis is acquired by the ingestion of contaminated soil or animal fecal material [16]. The animal herds at the outskirts of Sulaimaniyah make dung ubiquitous, and the dry climactic conditions throughout the warm months of the year may contribute to the windborne spread of organisms [16]. It is notable that five of the six patients with basidiobolomycosis were diagnosed in May, August and October, and that currently GI basidiobolomycosis is the most common deep fungal infection diagnosed in our community. It is also notable that cutaneous entomophthoromycosis, that is rare in arid climates where the great majority of GI basidiobolomycosis is reported, has not been seen in Sulaimaniyah.

In past years, sporadic cases of the biliary phase of fascioliasis have been recognized in the Sulaimaniyah governate, and in the autumn of 2007, one of the authors (TAH) conducted a study of local streams and identified *F. hepatica* rediae in 4% of Lymnaeid snails [38]. Rediae develop into tadpole-like cercariae that leave the snail and encyst as metacercariae on vegetation in or near streams and rivers [24,27,39]. Naturally growing watercress is sold in Sulaimaniyah markets. It is a favourite vegetable for human consumption with both wild and commercial products being common sources of infection [27,39]. Studies of hyperendemic fascioliasis in the South American Andes indicate that an increased risk of livestock infection and human transmission can be anticipated when greater numbers of cattle become concentrated around limited water sources [24]. As part of our current study, we found that 27% of cattle being processed at the regional slaughter house were infected with *F. hepatica*. The growth of Sulaimaniyah into areas still used for livestock may have exacerbated a generally low endemic level into one exposing increased numbers of humans to infection.

The acute phase of *Fasciola hepatica* responds well to a single course of triclabendazole [40]. Although triclabendazole has not been available in our pharmacies, symptoms and eosinophilia resolved with a 3 day course of 400mg of albendazole. Nevertheless, one of the patients treated with albendazole entered a chronic biliary phase of the disease two years after the initial diagnosis. Fatal fascioliasis occurs in livestock that are infected with large numbers of organisms, but this is extremely rare in humans for whom the principal manifestations are a prolonged low-grade febrile illness with some degree of anemia and hepatomegaly [28,41]

The reported cases of GI basidiobolomycosis nearly all had large or small intestinal obstruction that required surgery [19,29]. The recommended treatment is surgical removal of the infected tissues and an imidazole as anti-fungal agent with itraconozole being the most frequently recommended [16,19,36]. The organism tends to be resistant to amphotericin B [16,19,36]. In the review by Vikram et al. [19], eight deaths occurred among four-four patients (18%) with five of the fatalities being children [16-19,42-44]. Common features of the fatal cases were intestinal perforation and/or extensive abdominal spread of the disease [18,43]. Of the patients who died, only one was treated with itraconozole, 4 received no anti-fungal therapy, and 3 patients an amphotericin product. In our fatal case, the patient received amphotericin B as an anti-fungal agent and died as a result of intestinal perforation before the infected tissue could be resected.

The clinicopathologic findings for the two patients in which no diagnosis could be established were similar to those diagnosed with fascioliasis. The patients had isolated hepatic abscesses, abdominal pain, and eosinophila. Both patients were treated with albendazole for presumed toxocariasis more than a year before they were tested serologically. Following eradication of organisms, the ELISA serology for *Fasciola* is reported to become negative in 40% of patients at 6 months and more than 90% of patients after 12 months [45]. In contrast, anti-*Toxocara* IgG antibodies in patients treated for visceral toxocariasis usually remain elevated for years [46,47]. We recognize that an organism other than *F. hepatica* may have caused the two etiologically undiagnosed cases, but with the negative serology and inability to find larvae in the abscesses, we believe that a role for *Toxocara* has been reasonably ruled out and that these patients may have had fascioliasis with an attenuated antibody response.

In order to reach a correct diagnosis and determine the cause of an eosinophilic granulomatous abdominal abscess, pathologists, surgeons, and physicians should be aware of the different possible etiologies. A presentation as a GI mass must be distinguished from cancer, and the granulomatous inflammation surrounding the central abscess can be mistaken for tuberculosis. We have seen the eosinophilia of fascioliasis erroneously treated as an idiopathic hypereosinophilia syndrome with imatinib mesylate.

The fungus of basidiobolomycosis is readily identified if sufficient tissue is obtained but can be missed in mucosal biopsies as it was in two of our patients. The diagnosis of fascioliasis has historically been a problem. Serological testing is often unavailable, or an ineffective method may used. Before 2003, the sensitivity of serological tests was poor, but newer ELISA kits using ES or Fas2 antigens have an estimated sensitivity of 87-93% and are currently the recommended methods for diagnosing *F. hepatica*[33,34,40,45].

The gastrointestinal location of an eosinophilic granulomatous mass points to basidiobolomycosis, but rare examples of ectopic fascioliasis producing tumor-like colonic masses are reported [28,48,49]. In addition, hepatic abscesses are found in a significant proportion of patients with basidiobolomycosis [19]. It is not the location of eosinophilic granulomatous disease, but the identification of an *Entomophthorales* fungus that provides for the diagnosis of basidiobolomycosis, and the absence of fungus and a positive serological test for *Fasciola* that allows for the diagnosis of fascioliasis. Table 3 summarizes the pathological and serological findings for basidiobolomycosis, fascioliasis, and toxocariasis, the latter being included because it is considered a frequent cause of eosinophic granulomatous liver abscesses [9-11,13,46,50].

**Table 3:** Pathological and clinical laboratory findings in the differential diagnosis of abdominal eosinophilic granulomatous abscesses

	**Basidiobolomycosis**	**Fascioliasis**	**Toxocariasis**	**References**
Pathologic findings				
Anatomic site involved				
Intestine/colon mass	80% of patients	Rare, ectopic	Not reported	[16,19,46,48,49]
Liver abscesses	Localized, 30% of patients	Diffuse more common than localized.	Diffuse with VLM, localized uncommon	[9-11,13-15,19,28,35,46]
Microscopic findings				
Granulomatous inflammation	Present	Present	Present	[12-16,19,28]
Eosinophils	Present	Present	Present	[12-16,19,28]
Charcot-Leyden crystals	Not prominent	Numerous	Present	[12-16,19,28]
Organisms in tissues	Pauci-septate hyphae with Splendore-Hoeppli reaction required for diagnosis	Very rare, if present ova more common than flukes	Larvae 30% of cases	[12-16,19,28,46,50]
Clinical laboratory findings				
Eosinophilia	76% of patients, usually not very high	Consistently positive in acute stage	Consistently positive with VLM	[12-16,19,28,46,47,50]
Serology				
Before treatment	Immunodiffusion positive 50% of patients, not routinely available.	ELISA 87-93% sensitivity, 98% specificity for acute stage	ELISA 78% sensitivity, 98% specificity for VLM	[16,19,33,47,50]
After treatment	Unknown	> 90% negative at 1 year.	Positive for years after VLM	[16,19,33,45-47]

The identification of disease at surgery uncovers only the most severely affected patients. Serological studies for *F. hepatica* in the urban and rural populations of Sulaimaniyah as well as the examination of livestock are currently in progress to determine the level of the endemic situation. The clustering of basidiobolomycosis and *F. hepatica* has not been previously reported. The epidemiology of *F. hepatica* has been thoroughly studied but factors predisposing to basidiobolomycosis are poorly understood [16,20-25,30]. We believe Sulaimaniyah may provide an opportunity to identify conditions that contribute to human outbreaks of both diseases.

## Conclusions

Eosinophilic granulomatous abscesses unrelated to Langerhans histiocytosis are relatively rare human diseases of often obscure etiology. The city of Sulaimaniyah in the Eastern Kurdish region of Northern Iraq has experienced an outbreak of 14 of these lesions over a period of 40 months. Isolated liver abscesses attributable to *F. hepatica* were found in 6 of the 14 patients with the parasite also being identified in local cattle and as an endemic infestation of snails in local streams. Ulcerating GI abscesses caused by a Zygomycete fungus consistent with basidiobolomycosis occurred in 6 of the 14 patients. These two diseases have not been previously reported as occurring at the same time in the same region. The mode of transmission of basidiobolomycosis to humans is unknown, but the emergence in Sulaimaniyah together with fascioliasis supports the suggestion that basidiobolomycosis may be transmitted by the feces of grazing animals [16].

### Limitations

The study is observational and descriptive. As a descriptive study, it presents a relatively small number of patients that may bias generalizations regarding treatment, outcomes, and general manifestations of the diseases. The study is further limited by the lack of fungal cultures for the organism identified by histology as probable basidiobolomycosis.

## Abbreviations

GI: Gastrointestinal;ELISA: Enzyme linked immunoabsorbent assay;F. hepatica: Fascilola hepatica;PAS: Periodic acid Schiff

## Competing interest

The authors declare that they have no interests that compete with any of the contents of the manuscript.

## Authors’ contributions

HAH, RAM, NGR, and BEN contributed to data collection and analysis. HAH and RAM wrote the first draft of the manuscript. RMR, KHK, and TAH contributed patient information and revision of the manuscript. ABF and JG consulted on the case material and identified the GI disease as basidiobolomycosis. MDH, ABF, and JG helped write the several versions of the manuscript. The final version of the manuscript was edited and approved by all authors.

## Pre-publication history

The pre-publication history for this paper can be accessed here:

http://www.biomedcentral.com/1471-2334/13/91/prepub
